# Realizing high stretch ratio of flexible wavy circuit via laser carving

**DOI:** 10.1038/s41598-022-22594-2

**Published:** 2022-10-22

**Authors:** Jung-Hoon Yun, Adebisi Oluwabukola Victoria, Maenghyo Cho

**Affiliations:** 1 Department of Mechanical and Automotive Engineering, Kongju National University, 1223-24, Cheonan Daero, Seobuk-gu, Cheonan-si, Chungnam 31080 Korea; 2 Department of Future Convergence Engineering, Kongju National University, Cheonan, Korea; 3 Department of Mechanical Engineering, Seoul National University, Seoul, Korea

**Keywords:** Electrical and electronic engineering, Mechanical engineering

## Abstract

Stretchable wavy circuit is an essential component in flexible devices, which have wide applications in various fields. In the industrial field, the stretching ability of the circuit is a crucial factor for flexible devices. Therefore, this study proposes laser carving method to increase both stretch ratio and device resolution of the flexible device. The results obtained from the experiment and finite element analysis verifies that laser carving on the wavy circuit increases the maximum stretch ratio of wavy circuit. The obtained analytic model confirms that laser carving generates tilted section on the wavy circuit, and reduces the bending rigidity of the curvy point of the wavy circuit. The study also verified that laser carved groove induces crack propagation into vertical to the circuit direction, so that the laser carved wavy circuit is less likely to disconnect than uncarved wavy circuit. Due to the reduced bending rigidity and crack induce, the wavy circuit stretches more than the conventional uncarved wavy circuit.

## Introduction

Flexible devices have wide applications in the research fields of wearable devices^1,2^, soft robotics^3^, bio-sensors^4,5^, and energy harvesting^6^. On the other hand, in the industrial field, there are only few practical applications; for example, rollable/foldable display^7,8^. One of the promising materials for stretchable device is metal nano-wire composite, which is widely studied in elsewhere^9–17^, still structure issues remain in enhancing flexible devices. One of the representative structures used in flexible device is the island structure, which was suggested by Rogers et al. The rigid part of this structure is located in center and connected to a flexible circuit ^18–22^. Since the rigid part of this structure does not contribute to the stretch of the cell, the stretching ability of the circuit is a crucial factor for flexible devices. Various circuit structures such as kirigami^23,24^, helix^25^, and wrinkling^26–28^ have been developed to increase the maximum stretch ratio of the circuit; however, most of them required specialized fabrication techniques to achieve hardness in adopting mass production ^29^. Plane wavy structure, which is developed by Rogers et al. ^30–32^ is the most widely used structure incorporated with mass production of flexible device. However, it also had tradeoff between stretch ratio and device resolution^33^.

To increase both stretch ratio and device resolution, tilted wavy circuit design has been suggested^33^; however their specific methodology to adopt them with mass production has not been suggested yet. In this study, to increase stretching ability and device resolution, the laser carving method is suggested to fabricate tilted section on wavy circuit. To verify this study, the experimental and simulation methods have been introduced with mathematical analysis.

## Methods

### Experimental method

**Figure 1:**
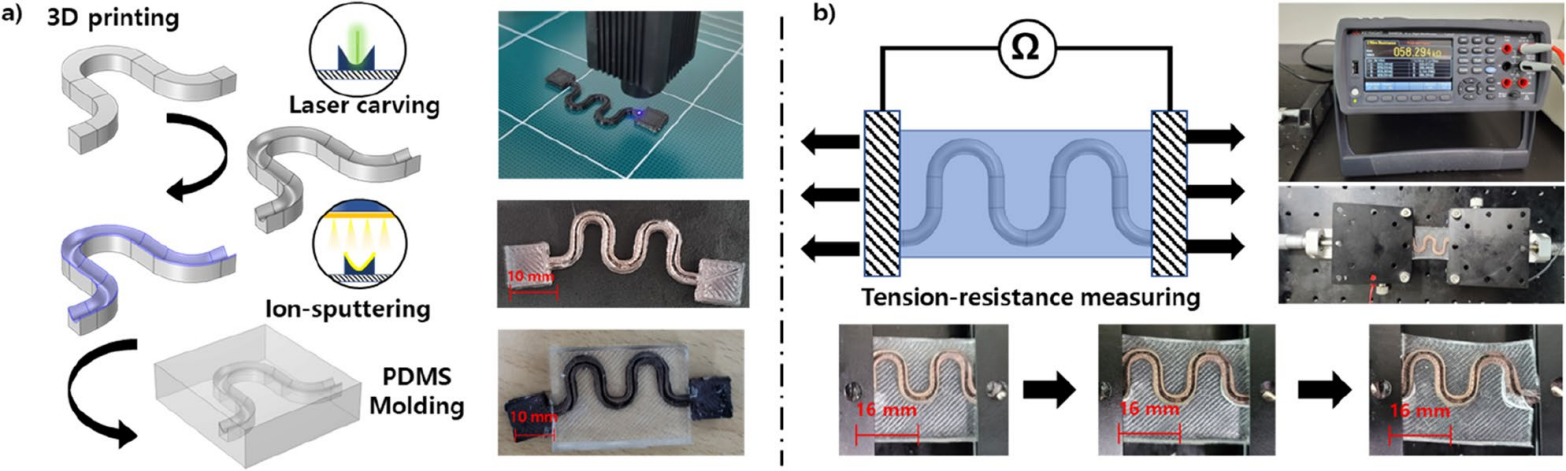
(**a**) Scheme for fabricating laser carved wavy circuit sample, and (**b**) resistance tension test.

Tilted wavy circuit sample has been prepared using both 3D printing (Moment 160) and laser carving methods. The circuit basis for laser carving has been prepared by printing wavy basis sample, as shown in Fig. 1a). To maximize the ablation of the laser on 3D printed base, black colored poly lactic acid (PLA) filament has been used for printing. Laser has been irradiated on the circuit base with their wavelength set to 365 nm, power set to 1750 mW, and spot time in each site set to 10 ms. The section for the wavy circuit has been illustrated in Fig. 1a). After laser carving, Au has been deposited on to the printed wavy circuit through ion-sputter (G20, GSEM), and their thickness has been measured as 2–5 μm. PDMS (Sylgard 184, Dow Corning) has been selected as the matrix surrounding wavy circuit, and their mixing ratio between major material and curing agent has been set from 10 to 1. The initially mixed PDMS-curing agent was placed in a vacuum chamber for 1 h at − 0.08 MPa from standard pressure to remove bubbles from mixing. The laser carved wavy circuit sample was made by pouring PDMS mixture into 3d printed mold, as shown in Fig. 1a). Thickness of the PDMS matrix is set as 4 mm.

To monitor the disconnection of the Au circuit, resistance of the wavy circuit has been monitored along with circuit tension, as shown in Fig. 1b). Regarding the point when the resistance of the circuit spikes as the disconnecting point, maximum stretch ratio of wavy circuit has been measured.

### Simulation method

**Figure 2:**
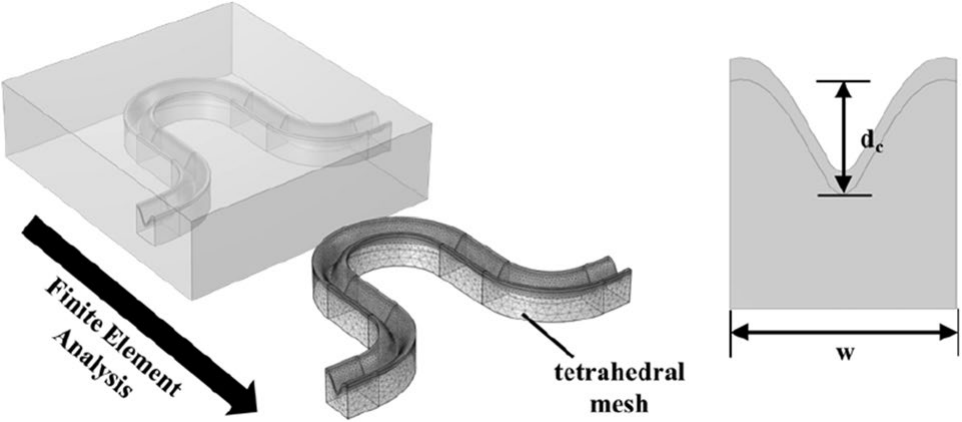
Scheme for modeling finite element method for laser carved wavy circuit and major simulation parameters ${d}_{c}$, $w$.

COMSOL has been used to perform finite element method to derive maximum stretch of wavy circuit, as shown in Fig. 2. MUMPS sparse solver in COMSOL is chosen to solve inverse problem. To increase the efficiency of the simulation and avoid non-positive Jacobian error, the thickness of the circuit layer was set as 0.4 mm. To measure the laser carving effect on maximum stretch ratio, carving depth, d_c_, has been used as a parameter for laser treatment. Considering multiple splines and layers in the model, tetrahedral mesh has been used for the simulation, and interface between different layer has been set as tie condition. Considering linearization, modulus and Poisson’s ratio for circuit basis (PLA), metal layer (Au), and matrix (PDMS) has been set as the values in Table 1. In order to make tension, one side of the circuit (including PDMS surface) is fixed, and prescribed displacement has been embedded on the other side of the wavy circuit.

**Table 1:** Mechanical property used in finite element method.

Domain	Young’s modulus (MPa)	Poisson’s ratio	Maximum stress (MPa)
Circuit base (PLA)^34^	1800	0.35	60
Metal layer (Au)^35^	50,000	0.4	500
Surrounding matrix (PDMS)^36^	0.750	0.49	5

## Simulation and experimental result

**Figure 3:**
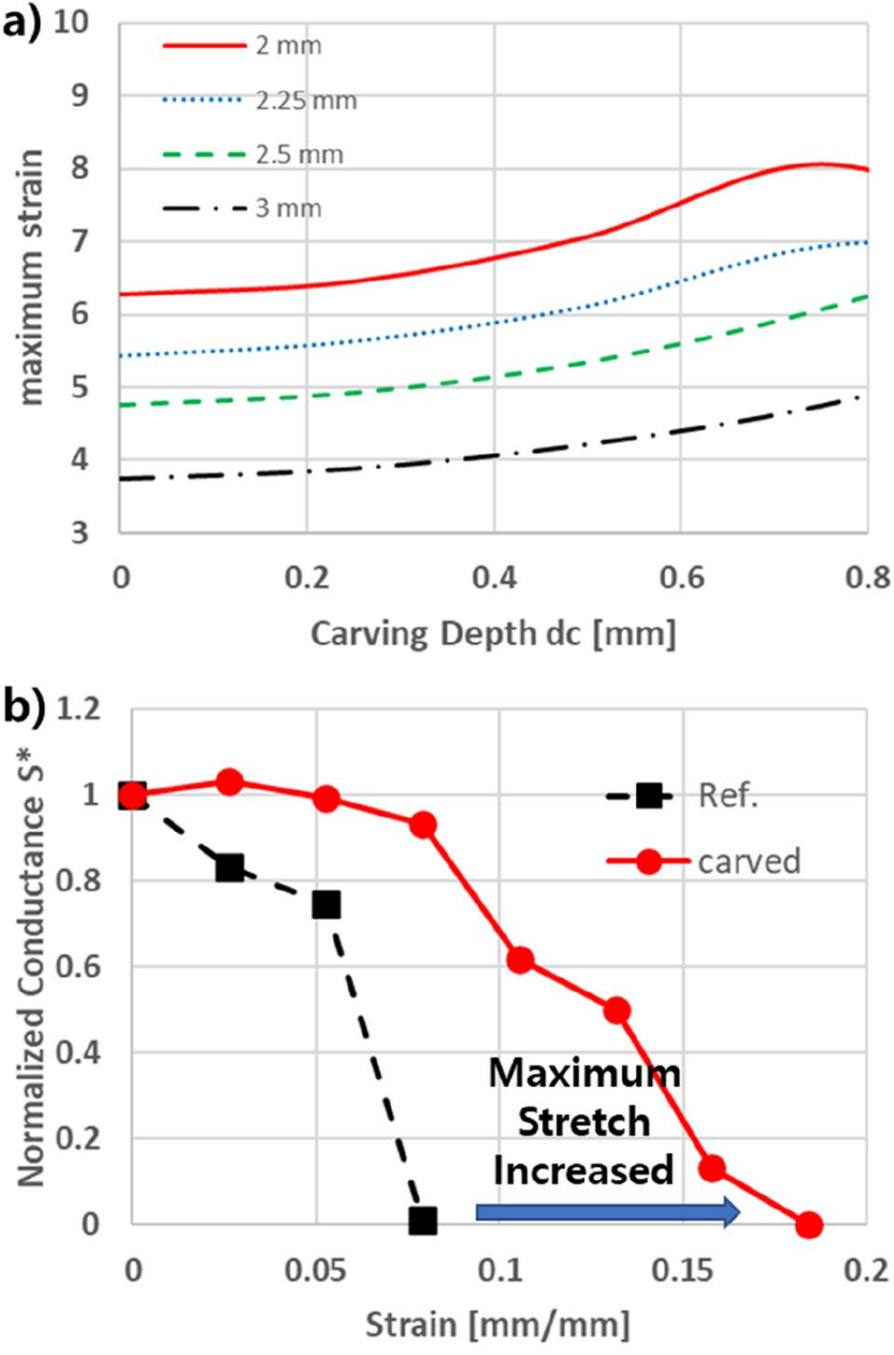
(**a**) Simulation result for maximum strain of laser carved wavy circuit in various carving depth and section width. (**b**) Experimental result for conductance changes along with stretch of wavy circuit.

Figure 3a shows the maximum stretch of wavy circuit with various carving depths, and width of wavy circuit. The simulation data implies that increasing the carving depth has advantage in increasing maximum stretch of wavy circuit in some extent; however, the simulation data also implies that excess laser carving will lessen the effect of increasing stretch ratio of wavy circuit.

Figure 3b shows the experimental result of conductance change in wavy circuit during the stretch. Conductance of the wavy circuit tends drop when the wavy circuit reaches maximum stretching point. The stretch-conductance data implies that maximum stretch of laser-carved wavy circuit shows better stretch ratio than plane wavy circuits. Specific data points for Figure 3 are described in supplementary file.

Table 2 shows the electric resistivity of wavy circuit and their arc length of the circuit in section of the wavy circuit. The data implies that increased surface area from laser carving increases conductivity of wavy circuit.

**Table 2:** Initial resistivity of wavy circuit.

Type	Uncarved		Carved	
	No sputtered	Au sputtered	No sputtered	Au sputtered
Section arc length (mm)	2.5	2.5	3.0311	3.0311
Resistivity (kΩ)	O.B*	2.898	O.B*	0.409

*Out of boundary > 100 MΩ.

**Figure 4:**
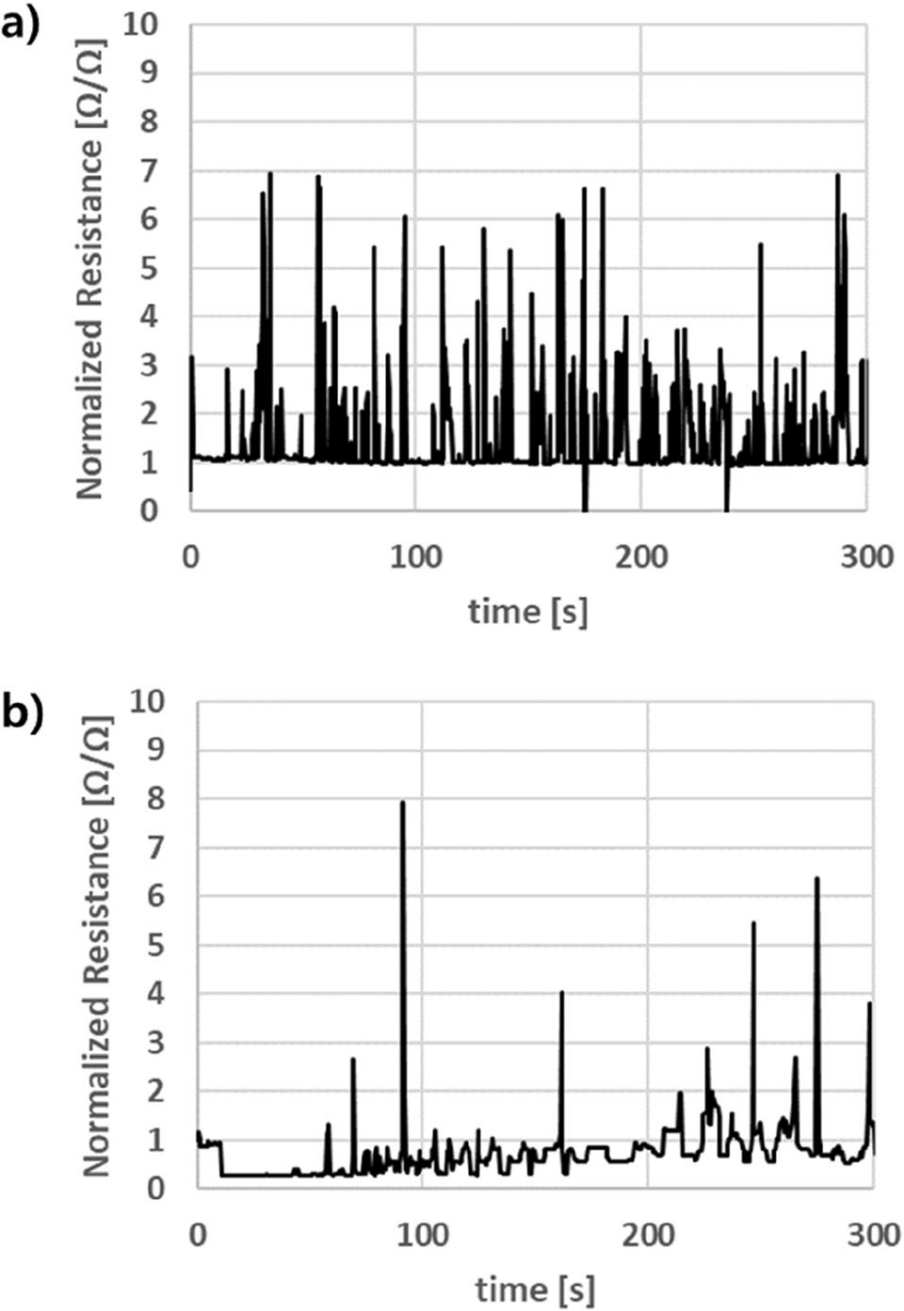
Change of resistance in wavy circuit during cyclic stretch (0.5 Hz, 10% amplitude) (**a**) without laser carving, and (**b**) with laser carving.

Figure 4 compares the cyclic tension-release property of carved wavy circuit with uncarved one. The amplitude for the tension cycle is set to 10%. When tension is applied to the circuit, the resistance tends to increase which returns to the initial state of resistance on removal of the tension.

The cyclic data implies that laser carved wavy circuit shows a lower resistance change compared to the uncarved one, which implies that the laser carved wavy circuit is relatively stable compared to plane wavy circuit in dynamic condition.

Both simulation and experimental results also indicate an increase in the maximum stretch ratio of the laser carved circuit while maintaining stable conductivity during tension.

## Discussion

### Effect of laser carving

**Figure 5:**
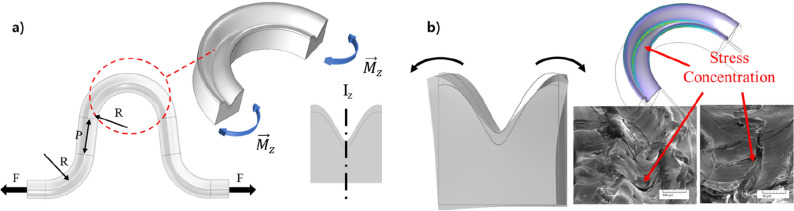
(**a**) Scheme for bending rigidity analysis in laser carved wavy circuit, and (**b**) off axial bending caused by the symmetric torsion of the tilted circuit section (scale bar for SEM: 500 μm and 50 μm).

Both simulation and experimental results imply that laser carving procedure increases the stretch of wavy circuit. Stress analysis of the simulation indicates that laser carving reduces the stress concentration in the curvy point. The decrease in stress concentration can be attributed to the decrease of the bending rigidity in the curvy point of the wavy circuit. With the induction of slope in section by laser carving, and deposition of circuit, the bending of curvy point becomes easier than that in plane one, which is expressed in the equations and Fig. 5a, as follows.

1 $$y = \frac{{4d_{c} }}{{w^{2} }}x^{2} - d_{c}$$

2 $$\begin{aligned} I_{z} & = I_{z,square} - I_{z,slope} = \frac{{w^{3} t}}{12} - \int_{A,slope} {x^{2} dA} \\ & = \frac{{w^{3} t}}{12} - \int_{{ - \frac{w}{2}}}^{\frac{w}{2}} {\mathop \int \nolimits_{{ - d_{c} }}^{0} } \left( {\frac{{y + d_{c} }}{{4d_{c} }}} \right) \cdot w^{2} dydx = w^{3} \cdot \left( {\frac{{4t - 3d_{c} }}{48}} \right) \\ \end{aligned}$$

Here, Eqs. (1) and (2) represent the slope of the laser carved surface, and bending rigidity of circuit section for each, respectively. $d_{c}$ and $w$ represents carving depth and width of the circuit section; $t$, $R$, and $P$ represent the thickness, radius of curvy point, and pitch of the wavy circuit for each, respectively, as shown in Fig. 5a. Assuming the bending of the curvy point as a beam, the bending angle of the curvy point can be expressed as follows:

3 $$M_{z} = \left\{ {R\left( {1 + \sin \theta } \right) + P} \right\} \cdot F = \kappa \cdot EI_{z}$$

4 $$\theta_{bending} \approx \mathop \int \nolimits_{0}^{\pi } 48 \cdot \frac{{\left\{ {\left( {R\left( {1 + sin\theta } \right) + P} \right) \cdot F} \right\}}}{{E \cdot w^{3} \cdot \left( {4t - 3d_{c} } \right)}}Rd\theta \approx \frac{48RF}{{E \cdot w^{3} \left( {4t - 3d_{c} } \right)}} \cdot \left\{ {R\left( {2 + \pi } \right) + \pi P} \right\}$$

Equation (3) represents the bending momentum applied in the section of the curvy point, and Eq. (4) represents the bending angle ($\theta_{bending}$) caused by stretch force $F$ in each end of the circuit. $E$ and $\theta$ represents modulus of the circuit and angle of the curvy point, respectively. Assuming small bending case, net strain caused by the bending of curvy point can be formulated as follows:

5 $$\epsilon \approx \frac{{P\theta_{bending} }}{2R} \approx \frac{24PRF}{{E \cdot w^{3} }} \cdot \left\{ {2 + \pi \left( {1 + \frac{P}{R}} \right)} \right\} \cdot \frac{1}{{\left( {4t - 3d_{c} } \right)}}$$

**Figure 6:**
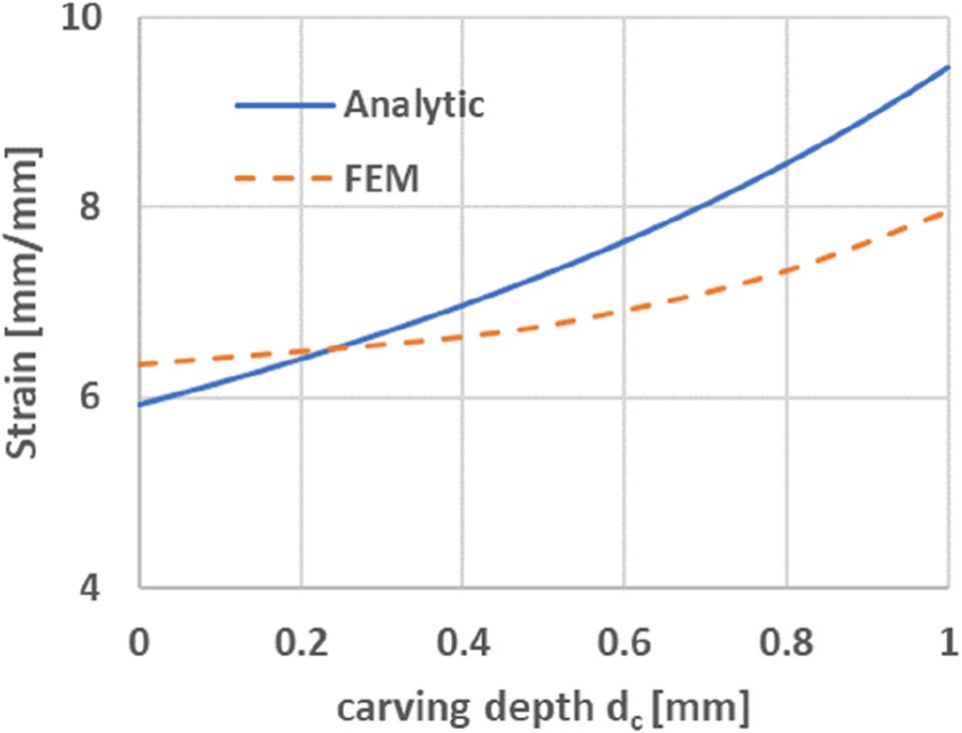
Analytic result of the maximum stretch on laser carved wavy circuit in 10 N tension.

Equation (5) represents net strain caused by stretch force ($F$). By setting the dimension similar to experimental conditions ($w = 2.5\;{\text{mm}}$, $E = 4.3\;{\text{GPa}}$, $P = R = 4\;{\text{mm}}$, $t = 2\;{\text{mm}}$) and setting $F = 10\;{\text{N}}$, maximum stretch of wavy circuit along with laser carving depth can be analytically illustrated, as shown in Fig. 6.

Stretch analysis for plane wavy circuit and density of the wavy circuit (inverse of the resolution) can be expressed as Eqs. (6) and (7)^33^:

6 $$\epsilon_{applied} = \frac{F}{{2Ew^{3} t}}\left\{ {P^{3} + 12\pi RP^{2} + 48R^{2} P + 6\pi R^{3} } \right\}$$

7 $$\rho = \frac{1}{{2R \cdot \left( {P + 2R} \right)}}$$

As stretch equation in laser carved circuit has laser carving parameter ($d_{c}$) in denominator, laser carved circuit can increase maximum stretch without increasing parameter pitch (P) and radius of curvy point (R). As increasing P and R decreases circuit density, in other worlds decreases device resolution, increasing laser parameter is better option for a wavy circuit to increase stretch ratio without sacrificing device resolution.

### Section distorting effect and vertical crack propagation

Increasing laser carving causes stress concentration and crack propagation, because the groove generated from laser carving become vulnerable to stress concentration caused by section distortion. The section width case of 2 mm, shown in Fig. 3a, implies that excessive carving depth induces structure failure during the stretch. FEM analysis also found that the tilted section in curvy point of wavy circuit tends to distort when stretch is induced. In the case of carved surface, there are two symmetric tilted section, and distortion tends to occur symmetrically as shown in Fig. 5b. Due to the symmetric distortion, curvy point in laser carved wavy circuit is also subjected to sectional bending, which induces stress concentration in the groove of the carved surface, as shown in Fig. 5b.

**Figure 7:**
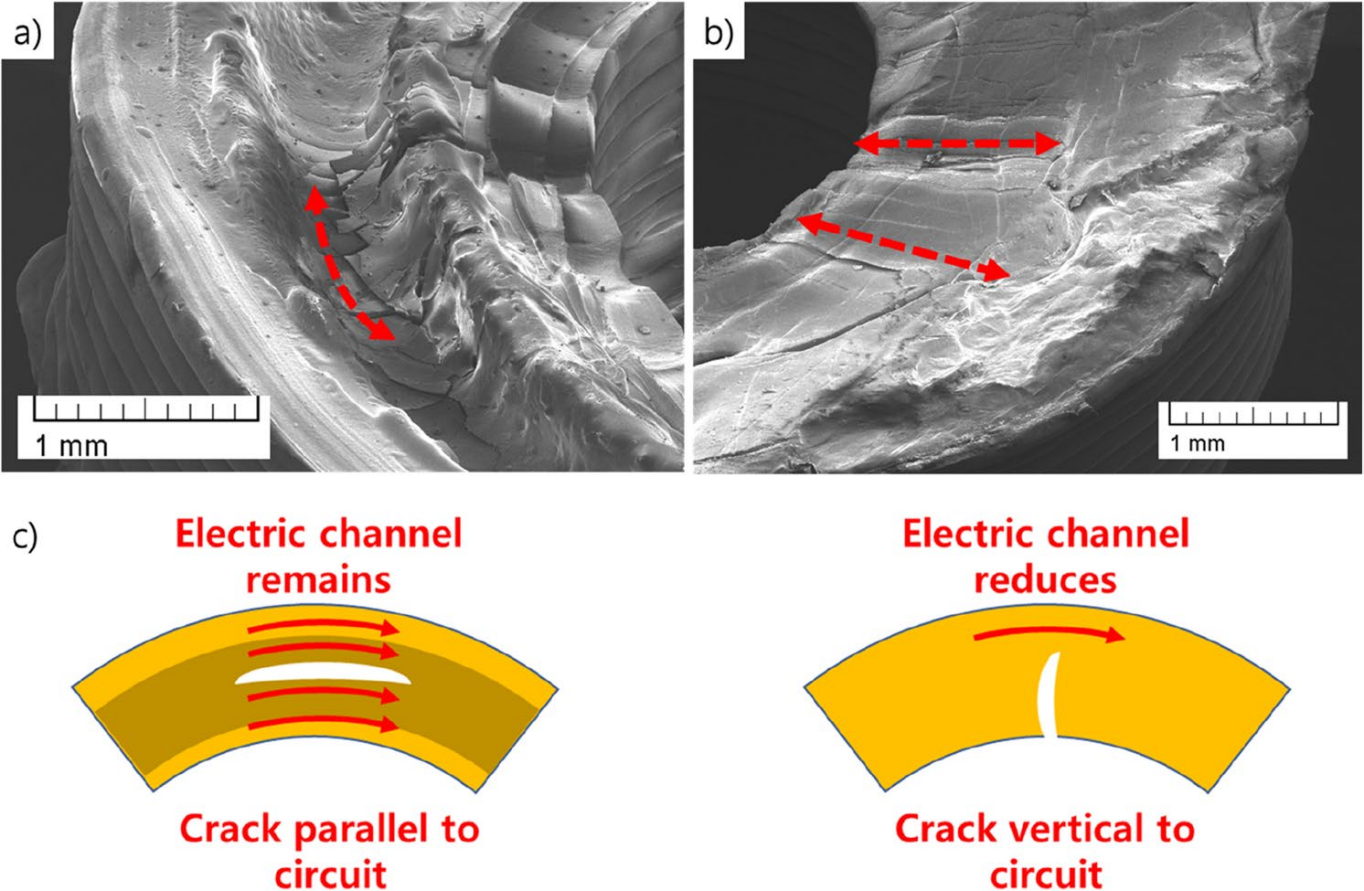
SEM image of crack propagation in (**a**) laser carved wavy circuit, and (**b**) uncarved wavy circuit. (**c**) Scheme for crack propagation in laser carved wavy circuit.

Nevertheless, actual disconnection of the wavy circuit during the stretch, shown in Fig. 3b, is quite differ to the simulation result in Fig. 3a. We assure the reason for the discrepancy between simulation and experiment is due to the direction of the crack propagation in wavy circuit. SEM image of crack propagation in wavy circuit (Fig. 7a,b) shows that the direction of crack propagation in laser carved circuit is parallel to circuit direction, which maintains electric conductive channel of wavy circuit even if the crack has been proceeded in some extent, as shown in Fig. 7c. We also assure the reason for slow drop of the conductance in laser carved wavy circuit is due to the parallel crack propagation, which lagged direct disconnection of the wavy circuit, as shown in Fig. 3b. The crack propagation in groove point of the laser carved wavy circuit implies that control of the groove point is essential in fabricating the circuit. If the curvature of the groove point of the laser carving is high, it will generate crack propagation in the end. Multiple laser carving, or extra heat treatment may be needed to control the groove point.

## Conclusion

Increasing the stretch ratio of wavy circuit is crucial for increasing the performance of flexible devices. However, conventional plane wavy circuit has various limitations when it comes to increasing the stretch ratio, because, in a wavy circuit, increasing the stretch ratio requires increasing the pitch, which eventually reduces the circuit density of the flexible device^22,24^. On the other hand, laser carved circuit has the effect of increasing the stretch of wavy circuit without tradeoff in device resolution, and it can induce crack parallel to the circuit direction to prevent disconnection of the circuit in some extent. Moreover, laser carving method can be easily incorporated with the conventional lithography or 3D printing technique to produce flexible devices, which is widely used in mass production of semiconductor OLED display and nano-materials^37–42^. Considering the advantages mentioned above; the laser carving method will significantly contribute to increasing the efficiency of producing flexible devices.

## Supplementary Information

Supplementary Information.

## Data Availability

The materials described in the manuscript, including all relevant raw data, will be freely available to any researcher wishing to use them for non-commercial purposes.

## References

[1] Kim T (2018). Versatile nanodot-patterned Gore-Tex fabric for multiple energy harvesting in wearable and aerodynamic nanogenerators. Nano Energy.

[2] Choi S, Lee H, Ghaffari R, Hyeon T, Kim DH (2016). Recent advances in flexible and stretchable bio-electronic devices integrated with nanomaterials. Adv. Mater..

[3] Zhu M, Do TN, Hawkes E, Visell Y (2020). Fluidic fabric muscle sheets for wearable and soft robotics. Soft Robot..

[4] Hammock ML, Chortos A, Tee BCK, Tok JBH, Bao Z (2013). 25th anniversary article: The evolution of electronic skin (E-Skin)—A brief history, design considerations, and recent progress. Adv. Mater..

[5] Zaghloul, M. E. MEMS, microsystems and nanosystems. In *Proceedings of the 2002 7th IEEE International Workshop on Cellular Networks and Their Applications*, 7492800.

[6] Zhang Z, Liao M, Lou H, Hu Y, Sun X, Peng H (2018). Conjugated polymers for flexible energy harvesting and storage. Adv. Mater..

[7] Hong JH (2017). The first 9.1-inch stretchable AMOLED display based on LTPS technology. J. SID.

[8] Li S, Peele BN, Larson CN, Zhao H, Shepherd RF (2016). A stretchable multicolor display and touch interface using photopatterning and transfer printing. Adv. Mater..

[9] Chang I (2014). Bendable fuel cell using highly conductive Ag nanowires. Int. J. Hydrog. Energy.

[10] Chang I, Park T, Lee J, Lee MH, Ko SH, Cha SW (2013). Bendable polymer electrolyte fuel cell using highly flexible Ag nanowire percolation network current collectors. J. Mater. Chem. A.

[11] Bang J, Jung Y, Kim H, Kim D, Cho M, Ko SH (2022). Multi-bandgap monolithic metal nanowire percolation network sensor integration by reversible selective laser-induced redox. Nano-Micro Lett..

[12] Kim H, Choi J, Kim KK, Won P, Hong S, Ko SH (2021). Biomimetic chameleon soft robot with artificial crypsis and disruptive coloration skin. Nat. Commun..

[13] Kim D (2020). Highly stretchable and oxidation-resistive Cu nanowire heater for replication of the feeling of heat in a virtual world. J. Mater. Chem. A.

[14] Hong I (2018). Study on the oxidation of copper nanowire network electrodes for skin mountable flexible, stretchable and wearable electronics applications. Nanotechnology.

[15] Won P (2019). Stretchable and transparent Kirigami conductor of nanowire percolation network for electronic skin applications. Nano Lett..

[16] Jung J (2019). Stretchable/flexible silver nanowire electrodes for energy device applications. Nanoscale.

[17] Hong S (2015). Highly stretchable and transparent metal nanowire heater for wearable electronics applications. Adv. Mater..

[18] Park S, Lee H, Kim YJ, Lee PS (2018). Fully laser-patterned stretchable microsupercapacitors integrated with soft electronic circuit components. NPG Asia Mater..

[19] Kang M, Byun JH, Na S, Jeon NL (2017). Fabrication of functional 3D multi-level microstructures on transparent substrates by one step back-side UV photolithography. RSC Adv..

[20] Abbasi R (2020). Photolithography-enabled direct patterning of liquid metals. J. Mater. Chem. C.

[21] Hashimi HA, Chaalal O (2021). Flexible temperature sensor fabrication using photolithography technique. Therm. Sci. Eng. Prog..

[22] Ma T, Wang Y, Tang R, Yu H, Jiang H (2013). Pre-patterned ZnO nanoribbons on soft substrates for stretchable energy harvesting applications. J. Appl. Phys..

[23] Choi WM, Song J, Khang DY, Jiang H, Huang Y, Rogers JA (2007). Biaxially stretchable “wavy” silicon nano-membranes. Nano Lett..

[24] Khang D-Y, Jiang H, Huang Y, Rogers JA (2006). A stretchable form of single-crystal silicon for high-performance electronics on rubber substrates. Science.

[25] Fabiano S, Facchetti A (2021). Stretchable helix-structured fibre electronics. Nat. Electron..

[26] Wang B, Bao S, Vinnikova S, Ghanta P, Wang S (2017). Buckling analysis in stretchable electronics. NPJ Flex. Electron..

[27] Ryu SY (2009). Lateral buckling mechanics in silicon nanowires on elastomeric substrates. Nano Lett..

[28] Khang DY (2008). Molecular scale buckling mechanics in individual aligned single-wall carbon nanotubes on elastomeric substrates. Nano Lett..

[29] Biswas S (2019). Integrated multilayer stretchable printed circuit boards paving the way for deformable active matrix. Nat. Commun..

[30] Su Y (2017). In-plane deformation mechanics for highly stretch-able electronics. Adv. Mater..

[31] Pan T (2017). Experimental and theoretical studies of serpentine interconnects on ultrathin elastomers for stretchable electronics. Adv. Funct. Mater..

[32] Huang X (2014). Stretchable, wireless sensors and functional substrates for epidermal characterization of sweat. Small.

[33] Yun J-H, Cho M (2020). Enhancing packing density and maximum elongation of 2D stretchable wavy circuit: Effect of section tilting. Mech. Adv. Mater. Struct..

[34] Soltani A, Noroozi R, Bodaghi M, Zolfagharian A, Hedayati R (2020). 3D printing on-water sports boards with bio inspired core designs. Polymers.

[35] Preiß E (2018). Fracture Toughness of Freestanding Metallic Thin Films Studied by Bulge Testing.

[36] Johnston ID, McCluskey DK, Tan CKL, Tracey MC (2014). Mechanical characterization of bulk Sylgard 184 for microfluidics and microengineering. J. Micromech. Microeng..

[37] Ko SH, Pan H, Grigoropoulos CP, Luscombe CK, Frechet JM, Poulikakos D (2007). All-inkjet-printed flexible electronics fabrication on a polymer substrate by low-temperature high-resolution selective laser sintering of metal nanoparticles. Nanotechnology.

[38] Ko SH, Pan H, Lee D, Grigoropoulos CP, Park HK (2010). Nanoparticle selective laser processing for a flexible display fabrication. Jpn. J. Appl. Phys..

[39] Hong S (2013). Nonvacuum, maskless fabrication of a flexible metal grid transparent conductor by low-temperature selective laser sintering of nanoparticle ink. ACS Nano.

[40] Hong S, Lee H, Yeo J, Ko SH (2016). Digital selective laser methods for nanomaterials: From synthesis to processing. NanoToday.

[41] Lee P (2012). Highly stretchable and highly conductive metal electrode by very long metal nanowire percolation network. Adv. Mater..

[42] Han S (2014). Flexible electronics: Fast plasmonic laser nanowelding for a Cu-nanowire percolation network for flexible transparent conductors and stretchable electronics. Adv. Mater..

