# Supplementary material for: Realizing high stretch ratio of flexible wavy circuit via laser carving
Source: Sci Rep. 2022 Oct 22;12:17745. doi: 10.1038/s41598-022-22594-2 (PMC9588079; doi:10.1038/s41598-022-22594-2)

<Supplementary>

Realizing High stretch ratio of flexible wavy circuit via laser carving

Jung-Hoon Yun[a,b,†], Adebisi Oluwabukola Victoria[b], and Maenghyo Cho[c]

[a]Department of Mechanical and Automotive Engineering, Kongju National University, Cheonan, Korea.
[b]Department of Future Convergence Engineering, Kongju National University, Cheonan, Korea.
c Department of Mechanical Engineering, Seoul National University, Seoul, Korea.
†Corresponding author:
Email: jhyun83@kongju.ac.kr
Address: 1223-24, Cheonan Daero, Seobuk-gu, Cheonan-si, Chungnam, 31080, Korea

---

Simulation Data for Figure 3-a)

strain data				
**c_d \ w**	**2 mm**	**2.25 mm**	**2.5 mm**	**3 mm**
**0**	6.3	5.4	4.8	3.8
**0.25**	6.5	5.6	4.9	3.9
**0.5**	7.1	6.1	5.4	4.2
**0.75**	8.1	6.9	6.1	4.7
**1**	7.0	6.9	7.1	5.6

Experimental Data for Figure 3-b)

Sample	Uncarved	
w	2.5	mm
t	2	mm
p	4	mm
r	4	mm
d_c	0	mm
L_0	38	mm

ΔL [mm]	R [kΩ]	ε	R*	S*
0	517.98	0	1	1
1	623.7	0.026316	1.204101	0.830495
2	693.41	0.052632	1.338681	0.747004
3	43790	0.078947	84.53994	0.011829

4	9.90E+37	0.105263	2.175374	0

Sample	Ref.			
w	2.5	mm		
t	2	mm		
p	4	mm		
r	4	mm		
d_c	0	mm		
L_0	38	mm		
ΔL [mm]	R [kΩ]	ε	R*	S*
0	517.98	0	1	1
1	623.7	0.026316	1.204101	0.830495
2	693.41	0.052632	1.338681	0.747004
3	43790	0.078947	84.53994	0.011829
4	9.90E+37	0.105263	2.175374	0

Sample	Carved	
w	2.5	mm
t	2	mm
p	4	mm
r	4	mm
d_c	0.75	mm
L_0	38	mm

ΔL [mm]	R [kΩ]	ε	R*	S*
0	19.14	0	1	1
1	18.514	0.026316	0.967294	1.033812
2	19.303	0.052632	1.008516	0.991556
3	20.524	0.078947	1.072309	0.932567
4	30.917	0.105263	1.615308	0.619077
5	38.346	0.131579	2.003448	0.499139
6	144.87	0.157895	7.568966	0.132118
7	9.90E+37	0.184211	3.045664	0

SEM Image of circuit section

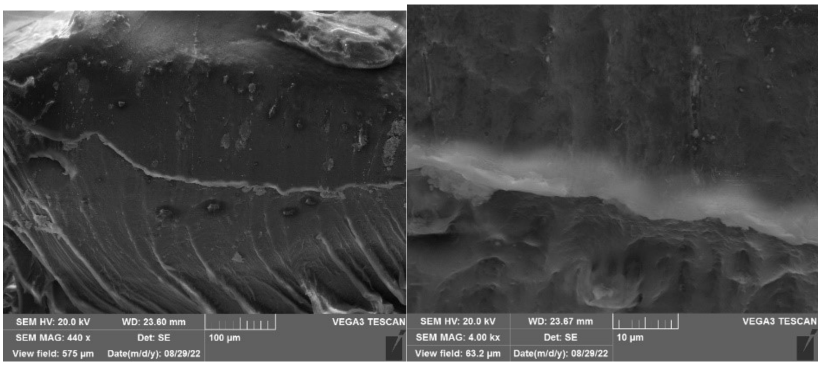

SEM Image of circuit surface

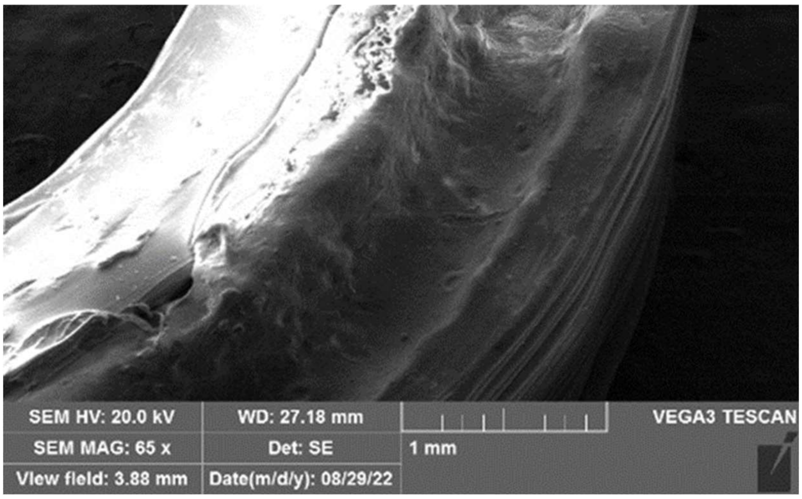

Supplement: Supplementary file 1 — Supplementary Information. [file 41598_2022_22594_MOESM1_ESM.pdf]
